# Microvascular reactivity is altered early in patients with acute respiratory distress syndrome

**DOI:** 10.1186/s12931-016-0375-y

**Published:** 2016-05-17

**Authors:** Diego Orbegozo Cortés, Lokmane Rahmania, Marian Irazabal, Carlos Santacruz, Vito Fontana, Daniel De Backer, Jacques Creteur, Jean-Louis Vincent

**Affiliations:** Department of Intensive Care, Erasme University Hospital, Université Libre de Bruxelles, Route de Lennik 808, B-1070 Brussels, Belgium

**Keywords:** Reactive hyperemia, Hypoxemia, Microcirculation, Prognosis, Mechanical ventilation

## Abstract

**Background:**

Acute respiratory distress syndrome (ARDS) is associated with vascular endothelial dysfunction. The resultant microvascular reactivity can be assessed non-invasively using near-infrared spectroscopy (NIRS) and a vascular occlusion test (VOT) and changes have been correlated with severity of organ dysfunction and mortality in other critically ill populations. We used NIRS to study the presence of microcirculatory alterations in patients with ARDS.

**Methods:**

We studied 27 healthy volunteers and 32 ARDS patients admitted to our intensive care department. NIRS measurements were performed within 24 h after diagnosis (Berlin definition). VOTs were performed by inflating an arm-cuff to a pressure greater than the systolic pressure for 3 min, followed by rapid deflation. The descending (Desc) and ascending (Asc) thenar muscle oxygen saturation (StO_2_) slopes were calculated. We compared data from volunteers with those from ARDS patients, from ARDS survivors and non-survivors, and from ARDS survivors who required <7 days ventilatory support (good evolution) with those who required >7 days support or died (poor evolution).

**Results:**

ARDS patients had lower StO_2_ values [75(67–80) vs 79(76–81) %, *p* = 0.04] and Asc slopes [185(115–233) vs 258(216–306) %/min, *p* < 0.01] than healthy volunteers, but Desc slopes were similar. The Asc slope was lower in the patients with a poor evolution than in the other patients [121(90–209) vs 222(170–293) %/min, *p* < 0.01], and in the non-survivors than in the survivors [95(73–120) vs 212(165–252) %/min, *p* < 0.01].

**Conclusions:**

In ARDS patients, microvascular reactivity is altered early, and the changes are directly related to the severity of the disease. The ascending slope is the best determinant of outcome.

**Electronic supplementary material:**

The online version of this article (doi:10.1186/s12931-016-0375-y) contains supplementary material, which is available to authorized users.

## Background

Acute respiratory distress syndrome (ARDS), defined using the Berlin criteria [1], is diagnosed in 25 to 30 % of patients admitted to the intensive care unit (ICU) who need mechanical ventilation for more than 24 h [2, 3], and is associated with high mortality rates [2–4] and long term sequelae [5, 6]. The complex pathophysiological alterations include functional and morphological alterations in the pulmonary endothelium [7], which may be correlated with the severity of the disease and outcomes [8–10].

Different options are available to study this endothelial dysfunction and the microcirculatory alterations that take place during ARDS. Invasive studies can be performed in animals to directly visualize the microcirculation using intra-vital video-microscopy [11]; levels of specific molecules (biomarkers) can be dosed in the plasma or in the bronchoalveolar fluid [12]; biopsies can be performed in selected cases [13]; or anatomo-pathological studies can be performed in the lungs of patients who die [7]. All these approaches have advantages and limitations. In clinical studies, the most invasive techniques can often not be applied and measurement of concentrations of single biomarkers does not reflect the complex underlying pathophysiology [14, 15].

Assessment of complex microvascular responses can now be performed non-invasively at the patient’s bedside using provocative tests of the microcirculation [16]. For example, using near-infrared spectroscopy (NIRS) it is possible to characterize the local muscle oxygen saturation (StO_2_) by applying the properties of oxy- and deoxy-hemoglobin to infrared light [17]. If the arterial supply to a region is transiently obstructed by a vascular occlusion test (VOT), the rate of decrease in the StO_2_ (desaturation) is a reflection of local oxygen consumption [18], and the rate of increase (re-saturation) after the VOT is related to the post-occlusive microcirculatory vascular hyperemia [19]. This dynamic test has been used to demonstrate that microvascular reactivity is altered in patients with sepsis [20], multiple organ dysfunction [21], trauma [22], acute heart failure [23], cardiac surgery [24] or after cardiac arrest resuscitation [25] and that the changes are not entirely explained by global hemodynamic variables. However, there are no data currently available in patients with ARDS. We, therefore, used NIRS to evaluate the presence, severity and prognostic implications of early microcirculatory alterations in patients with ARDS.

## Methods

This prospective, observational, non-invasive, physiological study was conducted in the 35-bed Department of Intensive Care of Erasme University Hospital (Brussels, Belgium). Institutional Ethical Committee approval was obtained, and all volunteers and patients (or their next of kin) provided written informed consent.

### Patients and volunteers

Healthy adult volunteers were recruited from the hospital staff. Adult ICU patients with ARDS were enrolled between October 2013 and June 2015 when they fulfilled the Berlin diagnostic criteria [1], including a PaO_2_/FiO_2_ ratio ≤ 300 on mechanical ventilation with a positive end-expiratory pressure (PEEP) of at least 5 cmH_2_O, and the presence of bilateral infiltrates on chest radiography, not explained by cardiogenic edema and in the presence of a risk factor for ARDS. Patients were stratified for severity using the PaO_2_/FiO_2_ ratio as recommended [1]. Patients were only included if a researcher was available to perform the NIRS measurements. All patients were managed according to current guidelines by a team of intensivists different from the researchers who performed the NIRS measurements.

### Protocol

The healthy volunteers were placed in a quiet, temperature controlled room within the department of intensive care, where they rested, comfortably seated for at least 15 min before the experiment. Heart rate (HR), respiratory rate (RR) and hemoglobin saturation by pulse oximetry (SpO_2_) were evaluated non-invasively with a Siemens SC 9000 monitor (Siemens, Erlangen, Germany). Non-invasive measurements of mean arterial pressure (MAP) were obtained on the opposite arm to that used for the NIRS measurements.

All patients were studied within 24 h after the diagnosis of ARDS was made. At the time when the NIRS VOT was performed, we recorded all measured hemodynamic and respiratory parameters from the monitoring systems. Biochemical and laboratory data closest to the time of the VOT were also recorded as was the presence or absence of any sedation. An APACHE II score [26] was calculated using the worst data during the first 24 h in the ICU, and a sequential organ failure assessment (SOFA) score [27] from the data present at the time of the NIRS measurements. We also recorded the duration of mechanical ventilation until definitive weaning from ventilatory support and calculated the ventilator-free days (VFD) at 28-days, giving a value of zero to non-survivors.

Survivors who required less than 7 days (arbitrarily selected) of respiratory support were classified as having a good evolution and those who required more than 7 days of mechanical ventilation or who died as having a poor evolution.

### NIRS measurements

**Fig. 1:**
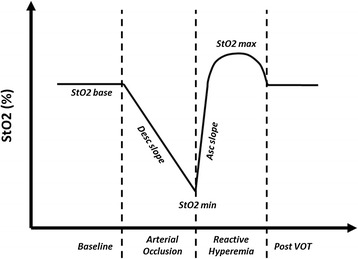
Schematic representation of tissue oxygen saturation (StO_2_) changes during a vascular occlusion test (VOT)

StO_2_ was measured using a near-infrared spectrometer (InSpectra 850 model, Hutchinson Technology, Hutchinson, Minnesota), with a 15 mm-probe attached to a fixed position on the thenar eminence (on the opposite hand from the arm with the invasive arterial line monitoring if present). Healthy volunteers and conscious patients were asked not to move their hand during the procedure. When the StO_2_ values had been stable for 1 min, we induced a VOT by rapidly inflating a sphygmomanometer cuff wrapped around the ipsilateral arm, to a pressure of 50 mmHg above the systolic arterial pressure for a period of 3 min (time threshold), followed by a rapid deflation. The StO_2_ curves were then analyzed using dedicated software (In Spectra Software Analyzer version 3.0., Hutchinson Technology). We measured the baseline, minimal and maximal StO_2_ (StO_2_base, StO_2_min and StO_2_max, respectively) and total hemoglobin index (THI base, THI min and THI max, respectively). We also computed the StO_2_ ascending (Asc) and descending (Desc) slopes (Fig. 1).

### Statistical analysis

Statistical analysis was performed using SPSS 22.0 (IBM, New York, NY) software. Dependent variables were assessed for normality of distribution using Skewness and Kurtosis tests and Q-Q plots. All values are presented as median values with percentiles (25–75 %), unless otherwise specified. Categorical data are presented as numbers of events and percentages. Comparisons between different cohorts were performed using a *t*-test, Mann–Whitney *U* test or Kruskal-Wallis H test as appropriate. Proportions were compared with a Chi square test or Fisher’s exact test as appropriate. A binary logistic regression was performed to identify the role of different variables to predict poor evolution, calculating the odds ratio (OR) and its respective 95 % confidence interval (CI). All variables that presented a p value < 0.1 during the univariate analysis were introduced into a multivariate analysis. We plotted the sensitivities and specificities in receiving operating characteristic (ROC) graphs and the area under the curve (AUC) was calculated for the different variables. A 2-sided p value less than 0.05 was considered as significant for all analyses.

## Results

Twenty-seven healthy volunteers were recruited (mean age 30 [range 27–31] years; 15 male) and 32 patients with ARDS (mean age 63 [range 48–71], 21 male). The main demographic, clinical and biochemical characteristics of the volunteers and patients are presented in Table 1. The characteristics of ARDS survivors and non-survivors are shown in Additional file 1: Table S1.

**Table 1:** Main characteristics of the healthy volunteers and the ARDS patients at the time of the VOT

VARIABLE	Volunteers (*n* = 27)	ARDS		
		Total (*n* = 32)	Good evolution (*n* = 16)	Poor evolution (*n* = 16)
Age (years)	30 (27–31)*	63 (48–71)	68 (46–78)	61 (51–66)
Male n(%)	15 (56)	21 (66)	12 (75)	9 (56)
Body mass index (Kg/m^2^)	23 (22–25)*	26 (23–29)	25 (24–28)	27 (22–30)
Mean arterial pressure (mmHg)	88 (83–91)	82 (75–89)	83 (80–93)	78 (72–87)
Heart rate (bpm)	74 (70–79)*	95 (81–104)	96 (81–105)	95 (83–102)
Respiratory rate (bpm)	12 (12–13)*	24 (19–30)	23 (18–30)	24 (20–31)
SpO_2_	98 (98–99)*	97 (95–99)	97 (95–99)	98 (96–99)
Hemoglobin O_2_ saturation (%)	-	97 (95–99)	97 (95–99)	98 (96–100)
Temperature (°C)	-	37.0 (36.3-37.5)	37.1 (36.6-37.6)	36.7 (36.1-37.2)
Sepsis n(%)	-	16 (50)	7 (44)	9 (56)
Primary ARDS n(%)	-	9 (28)	5 (31)	4 (25)
Trauma n(%)	-	4 (13)	2 (13)	2 (13)
Surgery n(%)	-	18 (56)	12 (75)	6 (38)
Chronic lung disease n(%)	-	5 (16)	1 (6)	4 (25)
Inspired O_2_ fraction (%)	-	50 (40–55)	50 (33–50)	50 (40–70)
Positive end-expiratory pressure (cmH_2_0)	-	6 (5–8)	7 (6–8)	5 (5–11)
PaO_2_/FiO_2_ ratio	-	172 (126–231)	181 (126–243)	172 (128–223)
pH	-	7.39 (7.34-7.44)	7.40 (7.34-7.44)	7.38 (7.33-7.45)
PCO_2_ (mmHg)	-	37 (36–44)	37 (36–43)	38 (35–53)
Lactate (mmol/L)	-	1.4 (0.8-2.5)	1.4 (0.8-3.2)	1.5 (0.9-2.3)
Creatinine (mg/dL)	-	1.1 (0.7-1.6)	1.1 (0.8-1.4)	1.0 (0.6-2.0)
Renal replacement therapy n(%)	-	4 (13)	1 (6)	3 (19)
Total bilirubin (mg/dL)	-	0.7 (0.5-1.1)	0.6 (0.5-1.1)	0.8 (0.6-1.3)
Platelets (x10^3^/μL)	-	129 (91–197)	143 (84–226)	121 (95–192)
Leukocytes (cells x10^3^/μL)	-	13.0 (9.4-16.7)	13.9 (11.9-16.2)	11.7 (7.6-17.9)
Hemoglobin (mg/dL)	-	9.6 (8.9-11.2)	9.6 (9.0-10.8)	9.7 (8.6-11.5)
Sedation n(%)	-	19 (59)	8 (50)	11 (69)
APACHE II score	-	22 (19–26)	21 (18–23)	25 (21–28)**
SOFA score	-	9 (7–13)	8 (5–11)	11 (7–15)
Norepinephrine (mcg/Kg/min)	-	0.01 (0.00-0.17)	0.00 (0.00-0.05)	0.07 (0.00-0.27)
Dobutamine (mcg/Kg/min)	-	0 (0–3)	0 (0–2)	0 (0–5)
ICU length of stay (days)	-	7.7 (4.4-13.7)	6.0 (4.0-7.7)	11.9 (7.3-20.6)**
Ventilator free days at 28 days (days)	-	21 (0–25)	25 (24–26)	0 (0–17)**
Mortality n(%)	-	8 (25)	0 (0)	8 (50)**

*significant difference at the *p* <0.05 level versus ARDS patients; **significant difference at the *p* <0.05 level versus good evolution

**Fig. 2:**
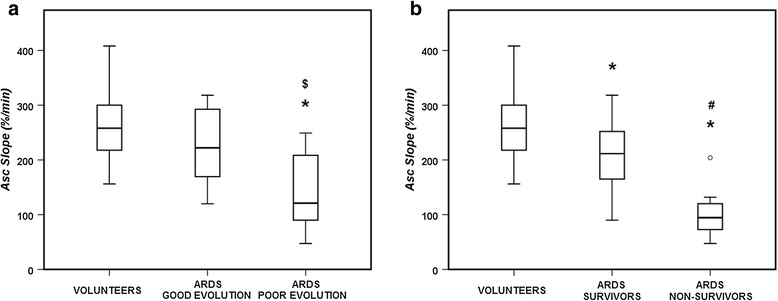
NIRS Asc slope in healthy volunteers and patients with ARDS according to evolution (**a**) and survival status (**b**). * = *p* < 0.05 compared with the volunteers; $ = *p* < 0.05 compared with patients with good evolution; # = *p* < 0.05 compared with the survivors

NIRS derived parameters are shown in Table 2. ARDS patients had lower StO_2_ values and Asc slope, but not Desc slope, than healthy volunteers. Patients with a poor evolution had a lower NIRS Asc slope than those with a good evolution (Fig. 2a), but StO_2_ and Desc slope values were not significantly different (Table 2). Likewise, non-survivors had a lower NIRS Asc slope than survivors (Fig. 2b). There were no differences in the Asc slope values according to the cause of ARDS (including sepsis and its severity) (Additional file 1: Figure S1 and S2).

**Table 2:** NIRS variables in healthy volunteers and ARDS patients

NIRS VARIABLE	VOLUNTEERS (*n* = 27)	ARDS				
		Total (*n* = 32)	Good evolution (*n* = 16)	Poor evolution (*n* = 16)	Survivors (*n* = 24)	Non-survivors (*n* = 8)
StO_2_ base (%)	79 (76–81)	75 (67–80)*	77 (72–80)	74 (60–80)	77 (73–80)	60 (59–76)*
THI base (au)	14.4 (12.8-15.4)	9.6 (6.8-10.8)**	9.7 (7.7-10.8)	9.0 (6.8-10.9)	9.8 (6.8-10.8)	7.9 (6.7-11.7)
StO_2_ max (%)	94 (90–94)	85 (76–90)**	87 (82–90)	84 (73–90)	87 (83–93)	73 (70–84)*
Desc slope (%/min)	10.3 (8.4-11.4)	9.6 (8.2-13.4)	11.3 (8.7-15.8)	8.8 (7.2-13.2)	11.3 (8.7-15.3)	8.1 (5.7-9.1)**
Asc slope (%/min)	258 (216–306)	185 (115–233)**	222 (170–293)	121 (90–209)**	212 (165–252)	95 (73–120)**

* = *p* < 0.05 and ** = *p* < 0.01, ARDS patients compared to healthy volunteers, poor evolution compared to good evolution, and non-survivors compared to survivors

**Fig. 3:**
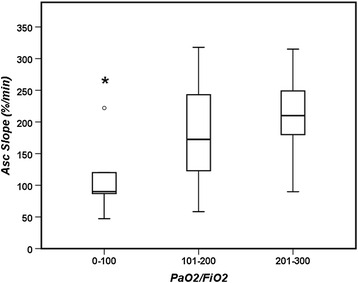
NIRS Asc slope in ARDS patients according to their PaO_2_/FiO_2_ ratio. * = *p* < 0.05 compared with the patients with a PaO_2_/FiO_2_ ratio between 201–300

There were no significant differences in the PaO_2_/FiO_2_ ratio between patients with a good or poor evolution, or between survivors and non-survivors (Tables 1 and Additional file 1: Table S1). However, ARDS patients with a PaO_2_/FiO_2_ ratio < 100 had a lower NIRS Asc slope than those with a PaO_2_/FiO_2_ ratio between 201 and 300 (Fig. 3).

**Fig. 4:**
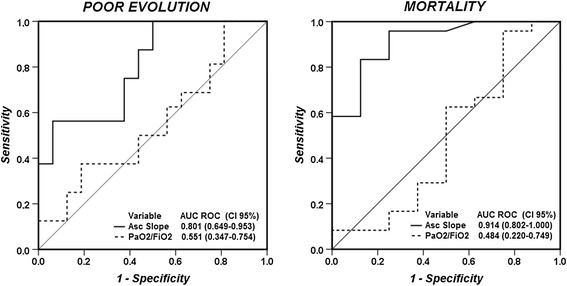
Receiver operating characteristic (ROC) curves for Asc slope and PaO_2_/FiO_2_ ratio to predict death (right panel) or poor evolution (death or more than 7 days of mechanical ventilation; left panel)

The NIRS Asc slope was a better predictor of poor evolution (and of mortality alone) than the PaO_2_/FiO_2_ ratio (Fig. 4). This was confirmed in the multivariate analysis in which the NIRS Asc slope was the most significant factor associated with a poor evolution during the ICU stay (Additional file 1: Tables S2 and S3).

## Discussion

The main finding of our study is that ARDS patients have altered peripheral microvascular reactivity as assessed using NIRS. This alteration is directly related to the severity of the disease characterized as death and/or prolonged need for mechanical ventilation.

The only published data on microcirculatory alterations in humans with acute respiratory failure assessed using non-invasive techniques were reported by our group in a small series of patients with acute lung injury due to Influenza AH1N1 [28]. These patients had diminished capillary density in the sublingual area, with a low proportion of perfused vessels and a reduced microvascular flow index. In the thenar muscle, post-occlusive reactive hyperemia was impaired, as manifest by low values of the Asc slope, similar to those found in septic patients in other studies [29–31]. Our current study extends these data to a larger and more heterogeneous population of patients with ARDS.

Half of the patients in our study had sepsis, but microcirculatory alterations detected by NIRS are not pathognomonic of septic conditions. Numerous studies have shown alterations of the Asc slope in septic populations, especially in the presence of shock [20, 29, 30], but low values of the Asc slope have also been observed in other acute conditions (normal values of 288 and 323 %/min have been reported [20, 22]): Gomez et al. reported values of 172 ± 103 %/min in trauma patients [22]; Petroni et al. reported values of 77 ± 42 %/min in patients with severe acute heart failure requiring veno-arterial extracorporeal membrane oxygenation (ECMO) support [23]; Morel et al. reported values of 144 (126–228) %/min 6 h after cardiopulmonary bypass in patients who had undergone cardiac surgery [24]; Donadello et al. reported values of 68 ± 32 %/min in resuscitated cardiac arrest patients after cessation of therapeutic hypothermia [25]; and we previously reported values of 186 ± 89 %/min in patients with circulatory shock of different etiologies [31]. In the present study, patients with septic shock had similar Asc slope values to patients with ARDS of non-septic causes. Although the mechanisms involved in the control of post-occlusive reactive hyperemia (NIRS Asc slope) have not been completely identified and are sometimes controversial [32, 33], it is well known that multiple vasodilator pathways (e.g., nitric oxide and prostaglandins) are necessary to obtain the transient increase in local blood flow [32] and complex inflammatory alterations will have been present in all the populations studied above [20, 22–25, 31]. Thus, the NIRS VOT is a functional test of the microcirculation, which measures its reserve (recruitability) under controlled ischemia, and involves multiple pathways.

Our findings suggest that microvascular endothelial dysfunction is present in the earliest stages of ARDS and is correlated with its severity. This concept fits well with the elevated concentrations of various pro-inflammatory molecules seen in patients with ARDS [8, 12, 34] and with autopsy findings revealing an initial pro-inflammatory and exudative phase [7, 35]. Dolinay and colleagues performed a series of experiments in mice and various cohorts of ARDS patients and showed that several inflammasome-regulated cytokines, including interleukin-18, play a major role in the development of ARDS [36]. Orfanos and colleagues demonstrated that severe ARDS was associated with decreased pulmonary capillary endothelium-bound angiotensin converting enzyme (ACE) function *in vivo* (an indirect marker of endothelial injury) [37]. More recently, Moussa et al. demonstrated that patients with ARDS in the context of sepsis had elevated numbers of circulating endothelial cells (a direct marker of endothelial injury), which were correlated with severity [10].

Early prognostication of outcome in ARDS is challenging. In large databases, the mortality rate is directly related to the severity of the alterations in gas exchange as expressed by the PaO_2_/FiO_2_ ratio, but the relationship is not very strong [38, 39], with an AUC of only 0.58 in a recent multicenter study [1]. We used a main combined outcome of death or need for prolonged mechanical ventilation which is essentially the opposite of ventilator-free days [40], but we also observed positive results when mortality was considered alone. Previous investigators have tried to predict similar combined outcomes, but they needed to use clinical data obtained during the first three days of hospitalization [41], rather than just on the first day as in the present study. Other groups have developed scores mixing various clinical and demographic data at admission, but these were not superior to the APACHE II score for predicting mortality [42]. We also observed that those patients who recovered rapidly had a similar Asc slope when compared with volunteers, despite the severe hypoxemia. Additionally, the NIRS Asc slope was the best predictor for death or more than 7 days on mechanical ventilation. Together these observations suggest that performing a NIRS VOT test on the first day of ARDS enables identification of patients at high-risk of poor outcome, potentially creating a target population for the testing of early interventions.

Our study has some limitations. First, it was a monocenter study and patient enrolment depended on researcher availability, although we included a heterogeneous sample of patients with different severities of ARDS. Second, we performed a single measurement and the time course of the Asc slope during subsequent days of treatment may provide additional information, as has been shown in septic patients [20]. Third, our control group was comprised of healthy younger individuals who may have different vascular reactivity to that of ARDS patients. However, we have previously reported that NIRS values are quite similar in control cohorts of patients and in healthy younger volunteers [20, 31]. Our comparison enables the whole spectrum from the normal values of health to those in severe ARDS patients to be considered. Finally, the sample size of our population was small, potentially explaining the non-significant differences in some variables, for example, the norepinephrine doses between patients with good and poor evolution (Table 1) or the NIRS Asc slope in the different groups of PaO_2_/FiO_2_ ratios, despite trends in the data. Nevertheless, the clinical relevance of the NIRS Asc slope was higher than that of other studied variables and this variable needs to be investigated further.

## Conclusion

In ARDS patients, there is an early, marked alteration in microvascular reactivity, which is directly related to the severity of the disease as reflected by the duration of mechanical ventilation and mortality.

## Additional file

Additional file 1: Table S1. Main characteristics of ARDS survivors and non-survivors. **Table S2.** Univariate analysis of variables to predict death or more than 7 days of mechanical ventilation. **Table S3.** Multivariable analysis of variables to predict death or more than 7 days of mechanical ventilation. **Figure S1.** Comparison of NIRS Asc slope in different groups of ARDS patients. **Figure S2.** Comparison of NIRS Asc slope in different groups of ARDS patients according to the presence or not of sepsis and septic shock. (DOCX 79 kb)
