# Supplementary material for: Microvascular reactivity is altered early in patients with acute respiratory distress syndrome
Source: Respir Res. 2016 May 17;17:59. doi: 10.1186/s12931-016-0375-y (PMC4869291; doi:10.1186/s12931-016-0375-y)

**MICROVASCULAR REACTIVITY IS ALTERED EARLY IN PATIENTS WITH**

**ACUTE RESPIRATORY DISTRESS SYNDROME**

Diego Orbegozo Cortés et al.

***Additional file***

**Additional file Table 1.** Main characteristics of ARDS survivors and non-survivors

| **VARIABLE** | **SURVIVORS**  **(n=24)** | **NON-SURVIVORS**  **(n=8)** | **P** |
| --- | --- | --- | --- |
| Age (years) | 63(44-72) | 63(58-66) | 0.83 |
| Male n(%) | 16(67) | 5(63) | 1.00 |
| Body mass index (Kg/m²) | 25(22-28) | 29(24-31) | 0.15 |
| Sepsis n(%) | 12(50) | 4(50) | 1.00 |
| Primary ARDS n(%) | 9(38) | 0(0) | 0.07 |
| Trauma n(%) | 4(17) | 0(0) | 0.55 |
| Surgery n(%) | 15(62) | 3(38) | 0.25 |
| Chronic lung disease n(%) | 3(13) | 2(25) | 0.58 |
| Mean arterial pressure (mmHg) | 84(80-93) | 75(72-78) | 0.01 |
| Heart rate (bpm) | 99(84-105) | 87(71-96) | 0.08 |
| Temperature (°C) | 37.1(36.6-37.5) | 36.3(34.8-36.7) | 0.02 |
| Inspired O_2_ fraction (%) | 50(40-50) | 48(40-80) | 0.72 |
| Respiratory rate (bpm) | 24(18-30) | 22(20-27) | 0.63 |
| Hemoglobin O_2_ saturation (%) | 97(95-99) | 97(96-99) | 0.83 |
| Positive end-expiratory pressure (cmH20) | 7(5-8) | 5(5-10) | 0.20 |
| pH | 7.40(7.34-7.46) | 7.38(7.35-7.43) | 0.54 |
| PCO_2_ (mmHg) | 38(36-46) | 37(35-43) | 0.56 |
| Lactate (mmol/L) | 1.2(0.8-2.1) | 2.3(1.5-5.6) | 0.04 |
| PaO_2_/FiO_2_ ratio | 172(126-225) | 191(116-259) | 0.90 |
| Creatinine (mg/dL) | 1.0(0.7-1.4) | 1.5(0.7-2.0) | 0.42 |
| Renal replacement therapy n(%) | 2(8) | 2(25) | 0.25 |
| Total bilirubin (mg/dL) | 0.6(0.5-1.0) | 0.9(0.7-2.4) | 0.22 |
| Platelets (x10³/µL) | 151(91-226) | 102(71-121) | 0.06 |
| Leukocytes (cells x10³/µL) | 13.0(10.5-16.2) | 13.0(7.6-23.6) | 0.70 |
| Hemoglobin (mg/dL) | 9.5(8.9-10.7) | 10.3(9.1-12.8) | 0.27 |
| Sedation n(%) | 12(50) | 7(87) | 0.10 |
| APACHE II score | 21(19-25) | 25(22-33) | 0.07 |
| SOFA score | 8(5-11) | 15(12-16) | <0.01 |
| Norepinephrine (mcg/Kg/min) | 0.00(0.00-0.08) | 0.27(0.03-0.60) | 0.01 |
| Dobutamine (mcg/Kg/min) | 0(0-2) | 3(0-8) | 0.13 |
| Ventilator-free days at 28 days (days) | 24(19-25) | 0(0-0) | <0.01 |
| ICU length of stay (days) | 7.7(5.1-14.0) | 7.3(3.6-11.9) | 0.66 |

**Additional file Table 2.** Univariate analysis of variables to predict death or more than 7 days of mechanical ventilation

| **Variable** | **OR** | **95% CI** | **P** |
| --- | --- | --- | --- |
| Age | 0.982 | 0.942-1.025 | 0.41 |
| Primary | 0.733 | 0.156-3.450 | 0.70 |
| Sepsis | 0.605 | 0.150-2.445 | 0.48 |
| PaO_2_/FiO_2_ ratio | 0.998 | 0.987-1.008 | 0.65 |
| Norepinephrine dose | 27.884 | 0.154-5045.675 | 0.21 |
| Surgical admission | 0.038 | 0.044-0.913 | 0.04 |
| APACHE II score | 1.165 | 0.998-1.361 | 0.05 |
| SOFA score | 1.154 | 0.964-1.383 | 0.12 |
| Asc slope | 0.982 | 0.970-0.995 | 0.01 |

OR: odds ratio; CI: confidence interval

**Additional file Table 3.** Multivariable analysis of variables to predict death or more than 7 days of mechanical ventilation

| **Variable** | **OR** | **95% CI** | **P** |
| --- | --- | --- | --- |
| APACHE II score | 1.218 | 0.981-1.551 | 0.07 |
| Asc slope | 0.981 | 0.967-0.996 | 0.01 |

OR: odds ratio; CI: confidence interval

**Additional file Figure 1.** Comparison of NIRS Asc slope in different groups of ARDS patients


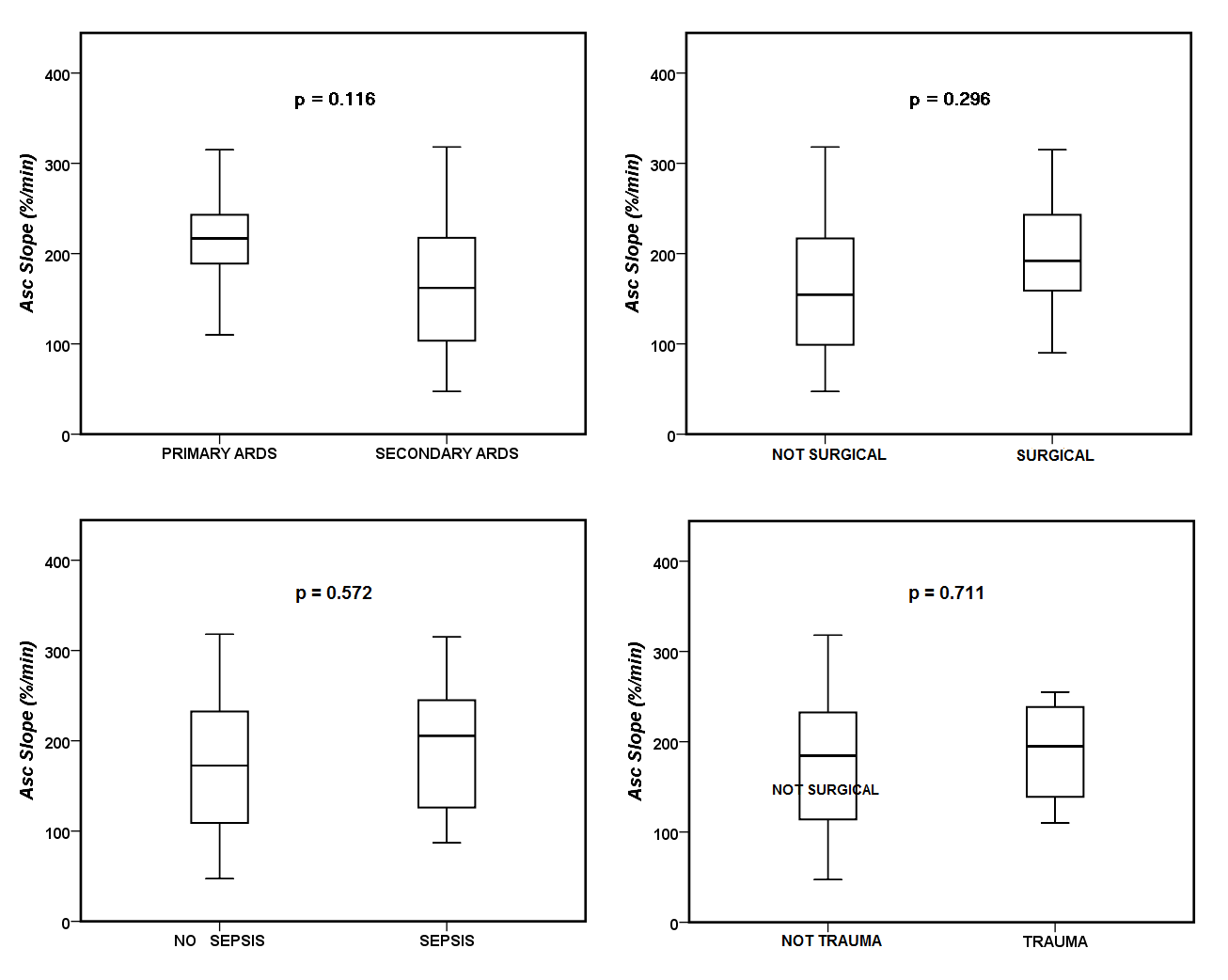


**Additional file Figure 2.** Comparison of NIRS Asc slope in different groups of ARDS patients according to the presence or not of sepsis and septic shock


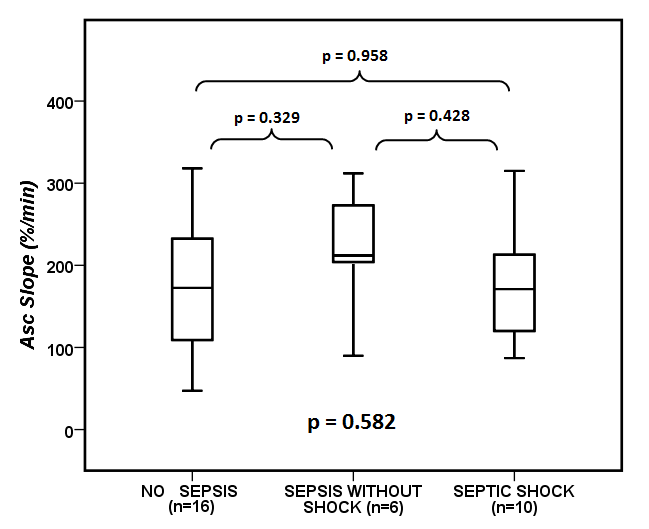

Supplement: Additional file 1: Table S1. — Main characteristics of ARDS survivors and non-survivors. Table S2. Univariate analysis of variables to predict death or more than 7 days of mechanical ventilation. Table S3. Multivariable analysis of variables to predict death or more than 7 days of mechanical ventilation. Figure S1. Comparison of NIRS Asc slope in different groups of ARDS patients. Figure S2. Comparison of NIRS Asc slope in different groups of ARDS patients according to the presence or not of sepsis and septic shock. (DOCX 79 kb) [file 12931_2016_375_MOESM1_ESM.docx]
